# Supplementary material for: Stacking Model‐Based Classifiers for Dealing With Multiple Sets of Noisy Labels
Source: Biom J. 2025 Mar 12;67(2):e70042. doi: 10.1002/bimj.70042 (PMC11898607; doi:10.1002/bimj.70042)
Supplement: Supplementary file 1 — Supporting Information [file BIMJ-67-e70042-s001.zip › density-based-ensemble-model-main/competitors_real_data/netlab-master/netlab-master/help/demtrain.htm]

Netlab Reference Manual demtrain


# demtrain

## Purpose

Demonstrate training of MLP network.

## Synopsis

```
demtrain
```

## Description

`demtrain` brings up a simple GUI to show the training of
an MLP network on classification and regression problems. The user
should load in a dataset (which should be in Netlab format: see
`datread`), select the output activation function, the
number of cycles and hidden units and then
train the network. The scaled conjugate gradient algorithm is used.
A graph shows the evolution of the error: the value is shown
`max(ceil(iterations / 50), 5)` cycles.

Once the network is trained, it is saved to the file `mlptrain.net`.
The results can then be viewed as a confusion matrix (for classification
problems) or a plot of output versus target (for regression problems).

## See Also

`confmat`, `datread`, `mlp`, `netopt`, `scg`

---

**Pages:**
Index


---

Copyright (c) Ian T Nabney (1996-9)
